# Admixture of anaesthetic drugs: editorial fit and safety are distinct concerns

**DOI:** 10.1007/s10877-026-01445-9

**Published:** 2026-06-01

**Authors:** Matthew Hart, Stephan Malherbe, Justin Skowno

**Affiliations:** 1 School of Child and Adolescent Health, Faculty of Medicine and Health, The University of Sydney, Sydney, Australia; 2 Department of Anaesthesia, The Children’s Hospital at Westmead, Sydney, Australia; 3 UBC Department of Anesthesiology, BC Children’s Hospital, Vancouver, Canada

## To the Editor,

We read with interest the recent editorial, “Admixture of anaesthetic drugs in the era of precision monitoring: a dangerous convenience” [1]. The editorial makes the case that a journal centred on precision monitoring should prefer studies in which hypnotic and analgesic agents are administered separately, thereby preserving independent titration and clearer physiologic interpretability. This position is an understandable one given the journal’s scientific emphasis. Our specific concern however, is with the editorial’s additional assertion that admixture is “dangerous”. This assertion appears to be based primarily on opinion pieces, is unsupported by data demonstrating lack of safety, and ignores the reported clinical application of admixtures (in particular propofol-remifentanil) across many thousands of anaesthetics globally each year.

Centres with decades of experience using propofol-remifentanil mixtures have reported complication profiles comparable to other anaesthetic techniques, without complications clearly attributable to the act of mixing itself [2, 3]. More recently, Bennion and colleagues published a prospective cohort study of single-syringe propofol-remifentanil TIVA in adults [4], adding contemporary prospective data to the discussion rather than evidence of obvious inherent danger. The editorial cites these studies, but then references several opinion and commentary pieces to justify it’s anti-mixing position, rather than clinical data demonstrating actual evidence of harm.

The evidence base isn’t mature enough to say that admixture is conclusively safe, nor that it should be specifically preferred where separate pumps, monitoring, and case characteristics allow separate administration and finer titration. It does suggest however, that the present evidence base is better described as supporting debate about precision, interpretability, and context of use than supporting the conclusion that admixture is dangerous per se.

A journal may reasonably decide that such techniques do not match its preferred methodological direction. That is a different proposition from implying that studies using admixture are unsafe by nature or scientifically unsuitable in all circumstances. For that reason, we would suggest a narrower and more defensible position: admixture may be less compatible with the aims of precision monitoring and mechanistic inference, and thus less aligned with the scope of a journal centred on those goals. However, that position should not be presented as though it were equivalent to a demonstrated safety verdict. Preserving that distinction would make the editorial policy clearer, more proportionate, and more consistent with the currently available clinical literature.

## Data Availability

No datasets were generated or analysed during the current study.
